# Imaging flow cytometry assays for quantifying pigment grade titanium dioxide particle internalization and interactions with immune cells in whole blood

**DOI:** 10.1002/cyto.a.23245

**Published:** 2017-09-20

**Authors:** Rachel E. Hewitt, Bradley Vis, Laetitia C. Pele, Nuno Faria, Jonathan J. Powell

**Affiliations:** ^1^ Biomineral Research Group, Department of Veterinary Medicine University of Cambridge, Madingley Rd Cambridge CB3 0ES UK; ^2^ Biomineral Research Group, MRC Elsie Widdowson Laboratory, Fulbourn Road Cambridge CB1 9NL UK

**Keywords:** imaging flow cytometry, darkfield, titanium dioxide particles, internalization, whole blood, monocytes, neutrophils, lymphocytes, membrane adherence, nanoparticles

## Abstract

Pigment grade titanium dioxide is composed of sub‐micron sized particles, including a nanofraction, and is widely utilized in food, cosmetic, pharmaceutical, and biomedical industries. Oral exposure to pigment grade titanium dioxide results in at least some material entering the circulation in humans, although subsequent interactions with blood immune cells are unknown. Pigment grade titanium dioxide is employed for its strong light scattering properties, and this work exploited that attribute to determine whether single cell–particle associations could be determined in immune cells of human whole blood at “real life” concentrations. In vitro assays, initially using isolated peripheral blood mononuclear cells, identified titanium dioxide associated with the surface of, and within, immune cells by darkfield reflectance in imaging flow cytometry. This was confirmed at the population level by side scatter measurements using conventional flow cytometry. Next, it was demonstrated that imaging flow cytometry could quantify titanium dioxide particle‐bearing cells, within the immune cell populations of fresh whole blood, down to titanium dioxide levels of 10 parts per billion, which is in the range anticipated for human blood following titanium dioxide ingestion. Moreover, surface association and internal localization of titanium dioxide particles could be discriminated in the assays. Overall, results showed that in addition to the anticipated activity of blood monocytes internalizing titanium dioxide particles, neutrophil internalization and cell membrane adhesion also occurred, the latter for both phagocytic and nonphagocytic cell types. What happens *in vivo* and whether this contributes to activation of one or more of these different cells types in blood merits further attention. © 2017 The Authors. Cytometry Part A Published by Wiley Periodicals, Inc. on behalf of ISAC.

**Abbreviations**
TiO_2_: titanium dioxide
PBMCs: peripheral blood mononuclear cells
ISX: Imagestream X
DF: darkfield
BDI: bright detail intensity
SSC: side scatter
FS: forward scatter
SLS: static light scattering
DLS: dynamic light scattering
NTA: nanoparticle tracking analysis
WB: whole blood
ICP: inductively coupled plasma
TEM: transmission electron microscopy

Human exposure to titanium dioxide (TiO_2_) particles via medical and consumer products is common place. Routes of exposure include the skin (e.g., sunscreens), the lungs (e.g., during painting), and the gastrointestinal tract (e.g., from processed foods, toothpaste, nutraceuticals, and pharmaceuticals) 1. Surgical titanium implants may also acquire oxide surfaces, which gradually wear releasing TiO_2_ particles. The research of our group with TiO_2_ has focused on the oral route in terms of quantifying human exposure, gastrointestinal uptake and, more recently, describing absorption into the circulation 2, 3, 4, 5, 6, 7. In common with other groups we have used inductively coupled plasma (ICP)‐based techniques to quantify titanium as well as reflectance microscopy, including darkfield microscopy, to detect TiO_2_ particles at the cellular level 7, 8. Recently, we showed that darkfield microscopy of blood films allows easy semiquantitative assessment of TiO_2_ in the human circulation if validated against ICP measures of Ti in the same samples 7. Nonetheless, a number of important questions remain unanswered in terms of the absorption of TiO_2_ particles into the circulation of humans, notably; Are the TiO_2_ particles associated with cells? If so, are these gut cells that have migrated or are they circulating blood cells? Aside from origin, are specific cell types targeted? What is the distribution of cells with internalized and outer membrane TiO_2_ adherence and finally, are TiO_2_‐targeted cells impacted according to markers of cell health and cell function? Clearly, analyses must be made at the single cell level. In this respect microscopy of blood films is not suitable. First, the dominance of red cells obscures the imaging and analysis of white cells, and while a red cell lysis step could be added to the sample preparation several important issues remain unresolved. In particular, a monolayer must be achieved to ensure adequate cell separation for attribution of particle interaction, cell phenotype, function, and cell health status (on a cell, by cell, basis). This limits the sampling volume to microliters. Moreover, no sufficiently advanced automated technique currently exists to determine accurately, by microscopy, multiple fluorescent markers of a monolayer of mixed cell types to yield detailed information on a cell‐by‐cell basis, let alone whether a TiO_2_ particle is inside or attached to a cell. Finally, data acquisition is manual, which reduces objectivity, is laborious, and further limits the sample size to likely, only hundreds of cells. The obvious solution is the use of flow cytometry, which objectively and rapidly, assesses high numbers of cells in mixed cell populations. Flow cytometry has been successfully applied to studying the biological significance of nano‐, sub‐micron‐, and micron‐sized materials in terms of their interactions at the cellular level. Multiple laser excitation possibilities and detectors allow the use of many fluorescent markers simultaneously such that extensive cellular characteristics can be identified. However, unlike microscopy, conventional flow cytometry does not provide images of cells to allow for any information on whether a particle is internalized within or adhered to a cell. It can only show particle association with the cell. To achieve all of the desirable analytical features noted above, the most decisive technique is imaging flow cytometry. It combines the advantages of the microscope with a flow cytometer. Inevitably, the microscopy is not as high resolution or flexible as that of a high end dedicated microscope and neither is its flow cytometric capability as flexible or high throughput as a dedicated high end flow cytometer. However, when it comes to the study of cell–particle interactions in complex (mixed cell) suspensions, it is truly fit for purpose: in particular for the assessment of particle or bacteria internalization and for quantitation of particle–cell localization 9, 10, 11, 12, 13, 14, 15. TiO_2_ is not fluorescent, but in pigment grade sizes, it is highly reflective with excellent light scattering properties. This light scattering property of TiO_2_ has been harnessed for the identification of TiO_2_‐bearing cells by flow cytometry and microscopy 16, 17, 18, 19, 20, 21, 22. We therefore sought to establish whether imaging flow cytometry would be a suitable technique for the analysis of low levels of TiO_2_ particles in whole human blood, especially providing information on cell targeting by the particles using light scatter rather than fluorescence as the signal. In particular, we asked whether imaging flow cytometry can rise to the challenge of detecting the relationship of TiO_2_ to cells in the complex environment of whole blood at levels of TiO_2_ at low parts per billion (i.e., so it is suitable for real life blood samples). Our approach was stepwise, first validating darkfield measures by imaging flow cytometry of TiO_2_‐cell interactions in peripheral blood mononuclear cells (PBMCs) against conventional flow cytometry side scatter (SSC) measurements 18. We then determined internalization and outer membrane adherence of TiO_2_ particles with cells residing within PBMC and whole blood by adapting conventional particle internalization methods for imaging flow cytometry 15. Finally, we demonstrated that the very low levels of TiO_2_ that are anticipated to occur in whole blood in vivo following oral challenge are discernable by this technique and cell targets can be identified.

## Methods

### Conduct of the Study

To initially examine the quantitation of TiO_2_ cellular association in peripheral blood immune cell populations, fresh leukocyte cones (also known as leukoreduction system chambers or filters) (*n* = 4) were purchased from the National Blood Service (Cambridge, UK), and PBMCs were isolated by density centrifugation using the separating medium Lymphoprep (Axis Shield Diagnostics Ltd, Dundee, Scotland). PBMCs were incubated with increasing concentrations of TiO_2_ and dual analyses performed as described in the following sections. For the investigation of TiO_2_ uptake in whole blood, fresh peripheral blood was obtained from healthy donors following informed consent. The study was approved by the local ethics committee; University of Cambridge, human biology research ethics committee, application HBREC.2015.10. TiO_2_ incubations and cellular analyses were carried out as described in the following sections.

### Preparation of TiO_2_ Particulate Solution

Food grade anatase TiO_2_ (product no 03970) was obtained in powder form from Sensient Colors Inc. (St. Louis, MO, USA). A stock dispersion was prepared by suspending the TiO_2_ at circa 1 g/l in distilled deionized water (DDW; 18 MΩ/cm), which was autoclave sterilized (121°C for 15 min). An aliquot of the TiO_2_ stock solution was then placed in a sonicating water bath for 20 min prior to use (i.e., for dilution, characterization, and/or cell culture applications).

### TiO_2_ Size Characterization

TiO_2_ powder was analyzed by transmission electron microscopy (TEM) and freshly prepared samples were analyzed by three independent methods, namely, static light scattering (SLS), dynamic light scattering (DLS), and nanoparticle tracking analysis (NTA). A stock of TiO_2_ (1 mg/ml) was sonicated for 15–20 min in a water bath sonicator prior to dilutions to 5 or 10 μg/ml in tissue culture medium (TCM) consisting of RPMI supplemented with 10% fetal calf serum and subsequent sizing.

### TEM

TiO_2_ powder was re‐suspended in ACS grade acetone and a drop of the solution placed on a 400‐mesh copper grid (Agar Scientific, Stansted, UK). After brief drying of the suspension at 50°C, TEM analysis was carried out at 80 kV with a FEI Philips CM100 TEM and images captured using a Deben AMT digital camera.

### SLS

SLS was performed on a Mastersizer 2000 with a Hydro 2000µP Micro Precision sample dispersion unit (Malvern Instruments Limited, Malvern, UK). The dispersion unit was run at 500 rpm, and care was taken to prevent bubble formation. Baseline correction was carried out with fresh TCM. A total of 100 μl of TiO_2_ stock were added to the 20 ml dispersion cell to achieve a final concentration of 5 µg/ml. Samples were analyzed in triplicates (refractive index: 2.493; absorption 0.1).

### DLS

DLS was performed on a Zetasizer Nano ZS (Malvern Instruments Limited) using Dispersion Technology Software 7.11. Particle suspensions were diluted 200‐fold in TCM (i.e., final concentration 5 µg/ml) before samples were measured in technical triplicates, applying refractive indices of 2.493 (absorption 0.1) for TiO_2_ particles and 1.33 for the dispersant.

### Nanoparticle Tracking Analysis (NTA)

NTA was performed on a Nanosight NS500 (Nanosight, Malvern, UK) using the NTA2.2 Analytical Software. Particle suspensions were diluted 200‐fold in TCM (i.e., final concentration 5 µg/ml) before samples were measured in technical triplicates for 30–60 s. Results were averaged after export into Microsoft Excel 2010.

### Zeta Potential

Electrophoretic mobility measurements of particle suspensions were performed by phase analysis light scattering on a Zetasizer Nano ZS using disposable zeta cells. Electrophoretic mobility of particles (measured voltage of 50.3 V) was converted into zeta potentials by the Dispersion Technology Software 7.11 using the Smoluchowski approximation, and a viscosity of 1.0031 cP and dielectric constant of 80.4 for the dispersant. Measurements were taken as three independent replicates.

Food grade TiO_2_ when imaged by TEM showed typical dry particle sizes of 100–200 nm, albeit with some smaller and larger sizes also present (shown in Supporting Information Additional file 1). When suspended in water or tissue culture media (TCM), at 5 μg/ml, mean ± SD zeta potentials, assessed by electrophoretic light scattering, were −20 mV ± 1 and −8.7 mV ± 0.2, respectively. In the same experiment, the aquated size of the suspended particles in TCM was also assessed by DLS (Supporting Information Additional file 1A) and the *Z* average (i.e., intensity‐weighted mean diameters derived from Cumulants analysis) was 300 nm. Sizing was re‐examined at 3 h, since particle suspensions are generally more reliably stable when the zeta potential is either above 30 mV or below −30 mV 23. Moreover, the re‐analysis at 3 h in TCM showed that size distribution remained relatively unaltered (*Z* average 339 nm; data not shown). At double the concentration in TCM (10 μg/ml TiO_2_), the *Z* average was 356 nm at 3 h and relative particle distribution remained similar to the other conditions.

Increases in particle size from the dry to aquated state, and then by a further 13–19% depending on concentration during three hours in TCM, were unsurprising due to the anticipated formation of a corona (e.g., hydration shell and interactions between the particle surface and TCM components such as protein) as well as a degree of agglomeration due to particle–particle interactions in solution 24.

DLS relies upon Brownian motion of nonsedimenting particles. Thus, while it is the most appropriate single technique for the analyses described above, it is still possible to “miss” (a) microparticles due to their sedimentation or (b) the true breadth of polydispersity in the nonsedimenting fraction due to masking of small nanoparticle signals by large nanoparticle signals (extent of light scattering by a given particle type is proportional to *d* ^6^, where *d* = diameter). In a population of particles, the smallest and largest fractions make up the “tails” of the distribution, by definition. Thus, for their analysis, techniques are generally chosen that can emphasize any signal from these tails thereby facilitating their detection. Hence, SLS, which commonly employs a stirred dispersion unit to prevent sedimentation and for which data are presented as “volume %,” enables a low number of larger particles to be detected in a high number of smaller particles because the former are maintained in suspension the output shows overall volume. Nanoparticle tracking analysis (NTA), in contrast, is best equipped to characterize polydisperse populations as it tracks particles individually and thus small particles are not “hidden” by the presence of larger, more light scattering particles. However, tracking is carried out on a horizontal focal plain and, as a consequence this technique under‐represents larger, sedimenting particle since these can drop out of the focal plain.

Here, SLS equipped with a dispersion unit, did reveal a less numerous second population of larger aggregates (ca. 1–10 µm; Supporting Information Additional file 1B) which were not easily observed by DLS, and skewed the average detected particles to larger sizes (circa 500 nm) as expected. Additionally, as anticipated, NTA revealed a dominant particle size consistent with the DLS data but also showed a second smaller peak of particles <100 nm in diameter (Supporting Information Additional file 1C), as previously reported for pigment grade TiO_2_ 25.

In summary, the most numerically frequent (as determined by NTA) aquated particle size of the TiO_2_ that is experienced by cells cultured in suspension is 150–300 nm but there is a range of sizes in suspension from <100 to circa 10,000 nm, with the larger sizes, which are not at all numerous, sedimenting out. In this way, experiments represented cell exposure to the natural size variance of food grade TiO_2_.

### Cell Isolation, Culture, and Fluorescent Antibody Staining

PBMCs, freshly isolated from leukocyte cones, were washed in RPMI and re‐suspended in tissue culture medium (TCM) consisting of RPMI supplemented with 10% fetal calf serum at 1 × 10^6^ cells/ml. After counting, cells were washed and re‐suspended in freezing medium, and stored frozen until use. Prior to experimentation, PBMCs were thawed, washed, and rested in TCM (supplemented with 2 mM l‐glutamine, 100 U/ml penicillin, and 100 μg/ml streptomycin; Sigma‐Aldrich, Dorset, UK) for 2 h. PBMCs were then incubated with TiO_2_ at concentrations indicated in the text (in the range of 0–10 μg/ml, which is 0–10,000 parts per billion [ppb]) for 3 h. Subsequent to TiO_2_ incubation, PBMCs were washed and re‐suspended in ∼200 μl ice‐cold phosphate‐buffered saline (PBS) containing 1% bovine serum albumin (BSA) wash buffer. Cells were then stained at the manufacturers' recommended staining concentration for 20 min on ice in the dark with either FITC or Alexa 488‐conjugated CD14 and CD3 PE Cy5 (all from BD Biosciences, Reading, UK). Single stain compensation tubes and unstained PBMC tubes, with and without TiO_2_, were also prepared at this time from PBMC samples to compensate for spectral overlap. After staining, PBMCs were washed again with ice‐cold PBS, 1% BSA, and re‐suspended in a small volume of PBS containing 2% paraformaldehyde (PFA) solution and placed on ice in the dark until acquisition.

### Whole Blood Assay

Fresh whole blood (WB) was collected from consenting healthy volunteers into tubes containing lithium‐heparin to prevent coagulation and briefly mixed within the tubes. WB was immediately transferred to Falcon tubes and a heparin solution (made from tissue culture grade heparin sodium salt dissolved in tissue culture grade H_2_O, both from Sigma‐Aldrich) was added to a final concentration of 0.5 mg/ml. After mixing, up to 200 μl of WB was then transferred to sterile FACS tubes for incubation with TiO_2_ at concentrations indicated in the text (0–10 μg/ml) for 24 h. Unchallenged cells (no TiO_2_ particles) were set up alongside as the negative control for each subject. Following incubation, 2 ml, 1× BD Pharm Lyse (BD Biosciences) solution at room temperature was added to the blood and immediately gently vortexed. Cells were then incubated in the dark for 15 min to allow RBC lysis and then centrifuged at 200*g* for 5 min. The supernatant was carefully aspirated, and cells were then washed twice in cold tissue culture grade dPBS. Cells were then washed with cold PBS containing 1% BSA and stained for 20 min on ice in the dark with cold PBS containing 1% BSA (FACS wash buffer) and the appropriate amount of antibody staining mix containing either FITC or Alexa 488‐conjugated anti‐human CD14 and CD16b PE (both BD Biosciences) at manufactures' recommended volumes. After staining cells were washed again with ice cold PBS, 1% BSA, and re‐suspended in a small volume of PBS containing 2% PFA solution and placed on ice in the dark until acquisition. Viability staining of neutrophil (CD16b^+^) and monocyte (CD14^+^) populations residing within whole blood at the end of the 24 h incubation period is shown in Supporting Information Additional file 2.

### Conventional Flow Cytometry

All flow cytometric investigations were performed using a CyAn ADP 9 colour analyser (Beckman Coulter, Ltd, High Wycombe, UK) equipped with 405 nm, 488 nm, and 642 nm solid‐state lasers and 11 detectors in standard configuration. Summit software was used for acquisition and analysis (Beckman Coulter). The machine was calibrated with single peak alignment beads (Spherotech), checking that coefficients of variation (CVs) resided within the target range (set by the manufacturer for the CyAn ADP) for each channel prior to acquisition of samples. A minimum of 400,000 events per sample were acquired for each sample. Samples were filtered through 35 μm nylon cell strainer mesh tubes (BD Biosciences) directly prior to acquisition. For data analysis, events were first plotted as forward versus side scatter (SSC) using SSC on a log scale and a large gate was drawn excluding debris. Cells were then further plotted for CD3, CD14, or CD16b versus forward scatter area to identify CD3^+^ T cells, CD14^+^ monocytes, or CD16b^+^ neutrophils. CD3^+^, CD14^+^, or CD16b^+^ gated cells were finally plotted as forward scatter (FS linear) versus SSC on a log scale and regions were drawn to identify SSC low and hi cells based on the no particle control for CD3^+^, CD14^+^, or CD16b^+^.

### Flow Cytometric Imaging

Flow imaging analysis was performed using an ImagesStream^X^ Mark I platform (Amnis‐Merck‐millipore) in standard configuration, equipped with 405 and 488 nm lasers for excitation and a 785 nm laser for a scatter signal with standard filter sets, multimagnification (20×/40×/60×) and extended depth of field. INSPIRE software (Amnis) was used for acquisition and IDEAS software (Amnis Seattle, WA, USA) for analysis. The machine was fully calibrated and passed all tests prior to each acquisition of samples using the machines calibration and test scripts. This included passing calibration scripts, which optimize camera synchronization, spatial offsets, and SSC calibrations for ×20, ×40, and ×60 objectives, dark current, core stage position, retro calibration, and horizontal laser calibrations. Single stain compensation tubes for each stain used, as well as an unstained tube and a TiO_2_ only tube, were prepared alongside test samples. Cells were filtered through 35 μm nylon cell strainer mesh tubes (BD Biosciences) directly prior to acquisition. A minimum of 30,000 events per sample were acquired. Compensation matrices were generated by running single stained particles or cells (i.e., single cell surface marker) and analyzed using IDEAS software. Data analysis is detailed in the results section. Briefly, cells were first plotted as area versus aspect ratio of the brightfield images and a single cell gate drawn, followed by a focused gate and CD14^+^, CD3^+^, or CD16b^+^ gates based on intensity of the respective fluorophore channels. Bivariate dot plots were then used to plot single focused CD14^+^, CD16b^+^, or CD3^+^ cells as the bright detail intensity of brightfield versus the bright detail intensity of darkfield and a region drawn to identify TiO_2_‐positive cells. The measurement of internalization was based on the Imagestream X (ISX) internalization feature, defined as the ratio of intensity measured inside a cell to the intensity of the entire cell. For the measurement of internalization, a mask eroded by 4 pixels from the brightfield cell image default mask was created and the bright detail intensity of darkfield (ch 6) measured within the eroded mask to enable the internalization score. Internalization high (hi) gates were drawn based on background values from the negative samples (no TiO_2_ controls). A full ‘working example’ of the gating and analysis strategy is shown in Supporting Information Additional file 3. To exclude the possibility that the increases in SSC (flow cytometry) and darkfield BDI (imaging cytometry) might have occurred as a consequence of cell toxicity and changes in cell size associated with apoptosis or necrosis, the imaging flow analysis was extended to include cell diameters of the CD14^+^ and CD3^+^ populations. No changes in cell diameter measurements were observed and both CD14^+^ and CD3^+^ populations remained a constant diameter irrespective of incubation with increasing concentrations of TiO_2_ throughout all donors, at all concentrations, as anticipated at these sub‐toxic concentrations 26, 27, thus this possibility was excluded (data is shown in Supporting Information Additional file 4).

### Statistics

Experiments examining TiO_2_ cellular association with multiple comparisons were assessed by one way ANOVA and post hoc analysis using Tukey's honestly significant difference (HSD) method with significance taken as *P* < 0.05. Experiments examining TiO_2_ cellular association at low dose compared to the control were assessed by paired, two‐tailed students *t* tests and significance taken at *P* < 0.025 as determined by the Bonferroni correction for two tests. Pearson's product–moment correlation coefficient was applied to evaluate the linear dependence between variables: correlation was considered significant if *P* values reached 0.001 or less (two‐tailed test).

## Results

### Quantitation of TiO_2_ Association by Imaging Flow Cytometry

To assess the ability of imaging flow cytometry to quantify TiO_2_ associating with cells within mixed peripheral blood cell populations using darkfield based measurements, we first carried out parallel analyses of TiO_2_ challenged PBMCs, isolated from the blood of healthy donors. PBMCs were incubated with increasing concentrations of TiO_2_ particles in tissue culture media for 3 h. Concentrations up to 10 μg/ml (10,000 ppb) were chosen having been previously reported as nontoxic doses 26, 27. Dual analyses were carried out using a conventional flow cytometer and run in parallel on an ISX imaging flow platform. This allowed direct comparison of the imaging flow data with data obtained from the same samples using established flow cytometric techniques for identifying TiO_2_ association with cells by increases in SSC intensity 18. PBMC is a mixed population of immune cells, consisting of T and B lymphocytes, monocytes, natural killer cells, and dendritic cells. CD14^+^ monocytes and CD3^+^ T lymphocytes were assessed for TiO_2_ association as the major phagocytic and non‐phagocytic immune cell types normally present within PBMC, respectively.

**Figure 1:**
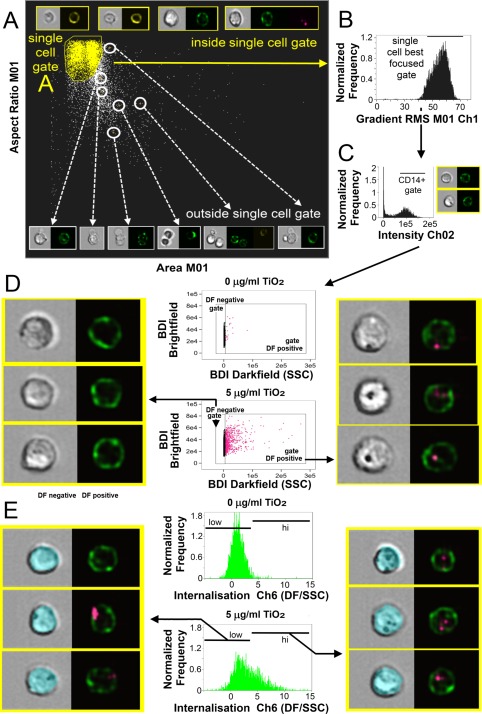
TiO_2_ localization and association with monocyte populations within PBMC. (**A**) For imaging flow analysis, area (size of the masked cells in μm^2^) versus aspect ratio (the ratio of the minor axis divided by the major axis) of the brightfield cell images are used to create an initial dot plot identifying cells of interest and excluding doublet and debris. Representative plot and selected image examples are shown. From the single cell gate cells in best focus were gated (using gradient RMS) shown in (**B**), followed by gating on CD14 fluorescence positives using fluorescence intensity histograms for CD14^+^ in Ch 02. (**C**) Single, focused gated CD14^+^ cells were then plotted as scatter plots using bright detail intensity (BDI) measurements for brightfield (vertical axis) and darkfield (horizontal axis). The bright detail intensity (BDI) feature computes the intensity of localized bright spots three pixels or less within the masked cell area of the image. A region is then drawn selecting TiO_2_ positive cells, shown in (**D**). Representative brightfield and merged darkfield (pink)/CD14^+^ fluorescence (green) image examples of the cells residing within gates are shown. Measurement of the percentage of CD14^+^ population with internalized TiO_2_ particles identified by increased darkfield BDI measurements were defined using the internalization feature and assigned an internalization score. Histogram plots of internalization scores for Ch6 (Darkfield/SSC) were used to create internalization hi and low gates within the CD14^+^ gated populations shown in (**E**), alongside masked (blue) brightfield and merged darkfield (pink)/CD14^+^ fluorescence (green) image examples. [Color figure can be viewed at wileyonlinelibrary.com]

For imaging flow cytometric analysis, gating strategies similar to those used for conventional flow cytometry are applied. Conventional flow cytometry generally first uses forward and SSC linear measurement scatter (dot) plots to identify cell populations of interest and exclude debris. For imaging flow analysis, area (the size of the masked cells in square microns) versus aspect ratio (the ratio of the minor axis divided by the major axis) of the brightfield cell images is used to create an initial dot plot to identify cell populations of interest as well as doublet and debris exclusion. As with conventional flow cytometry each “dot” represents a cell but for the imaging flow dot plot each “dot” additionally has a microscopic image. By clicking on the “dots” and examining the corresponding images an accurate single cell gate can be drawn. A representative plot and image examples are shown in Figure 1A. From the single cell gate further gates are drawn, starting with cells in best focus (using gradient RMS, which measures the sharpness quality of an image through the enumeration of pixel values, shown in Fig. 1B), followed by gating on fluorescence positives through fluorescence intensity histograms of channels of interest. For simplicity, the analysis of CD14^+^ phagocytic cells is shown in Figure 1. CD14^+^ (single, focused) gated cells were then plotted as scatter plots using bright detail intensity (BDI) measurements for brightfield (vertical axis) and darkfield (horizontal axis). The analysis is based on darkfield measurements, therefore darkfield BDI was the most discriminative parameter. However, since in single parameter histograms individual events (cells) are obscured due to binning, 2 ‐parameter scatter histograms are more useful for identifying individual cells and examining associated images in the IDEAS software. In fig 1D, the brightfield BDI was used as the vertical axis parameter but other image parameters could also have been used for this purpose. The bright detail intensity (BDI) feature computes the intensity of localized bright spots within the masked cell area of the image. In this analysis, BDI R3 was used, which computes the intensity of bright spots that are 3 pixels in radius or less. BDI scatter plots of the gated CD14^+^ allowed a region to then be drawn selecting CD14^+^ TiO_2_ positive cells, identified by increased BDI darkfield measurements, shown in Figure 1D alongside representative brightfield and merged darkfield (pink)/CD14^+^ (green) fluorescence image examples of the cells residing within the BDI darkfield positive and negative gates.

The measurement of internalized TiO_2_ darkfield signals relies on combining the application of two imaging cytometry features. First, imaging cytometers use a microscope objective to collect transmitted light, scattered light and emitted fluorescence 28. At the standard 40× objective used in these assays this provides a standard cross sectional image of each cell with a 4 μm depth of focus (for the PBMC titration experiments an extended depth of field was applied giving 16 μm focal range which allowed crisper fluorescent imaging throughout the depth of the cell image, but this is not necessary for a cross sectional image). This cross sectional representation of each cell allows the definition of the cell surface membrane at the outer perimeter of the cell image, as can be seen in Figure 1 where the location of cell surface associated CD14 (monocytes, green) can be seen. This cross‐sectional image is then utilized to create a custom mask, which provides information on a specific area of interest. For internalization, this is the area of the cell excluding the outer cell membrane, and so an internalization mask was created eroded by 4 pixels from the outer edge of the cell brightfield image. Each pixel has a size of 0.5 μm so creating an area of interest up to the equivalent to 2 μm in from the cell surface membrane. This excludes darkfield signals associated with TiO_2_ at the cell surface. Masks, once applied, are unique to every cell: examples can be seen on the images shown in Figure 1E. Measurement of the percentage of CD14^+^ (or CD3^+^) population with internalized TiO_2_ particles, identified by increased darkfield BDI measurements, was defined using the internalization feature (defined as the ratio of intensity inside the cell to the intensity of the entire cell) and assigned an internalization score, with higher scores indicating internalization. Histogram plots of internalization scores for Ch6 (Darkfield/SSC) were used to create internalization hi and low gates within the CD14^+^ gated populations (Fig. 1E). As for all aspects of imaging flow analysis, examination of associated cell masks and cell images greatly assists in setting accurate gates and improves overall accuracy 28.

**Figure 2:**
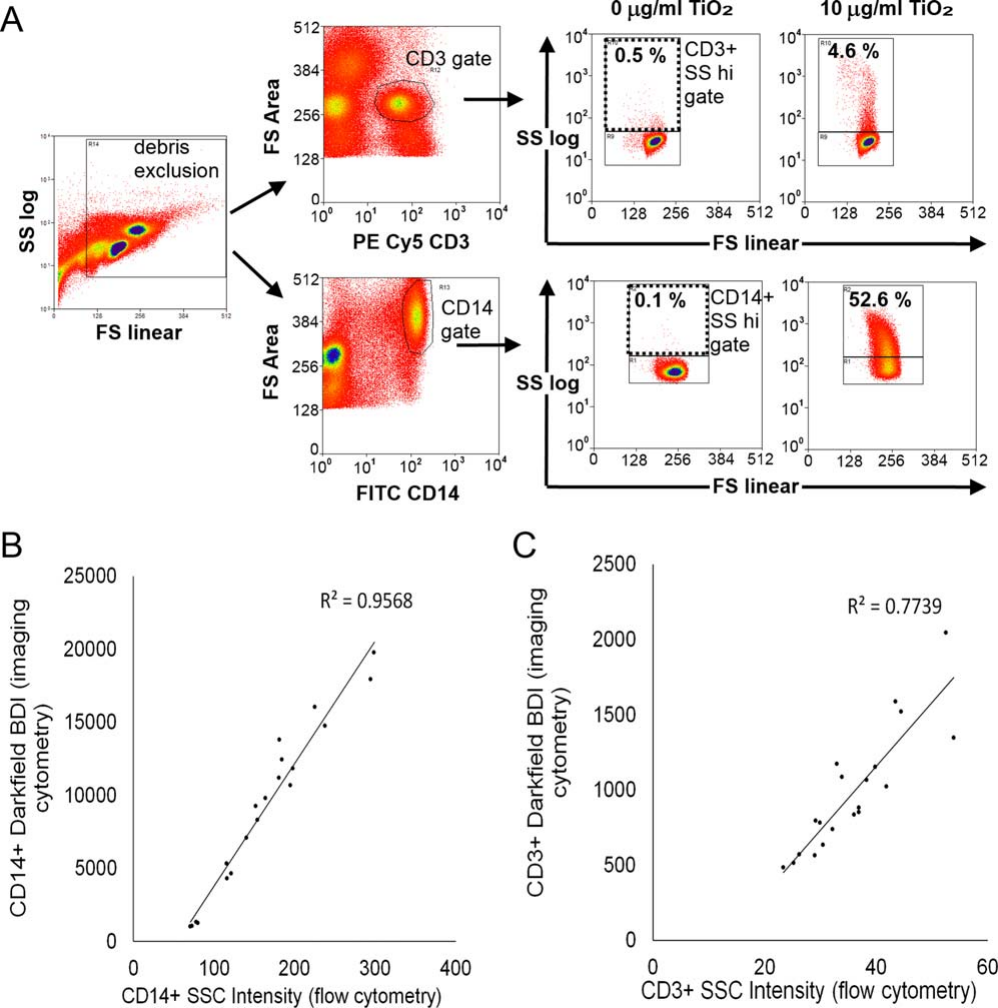
Conventional flow cytometry quantitation of monocyte and lymphocyte populations within PBMC association with TiO_2_ and correlation between conventional and imaging flow techniques. **(A**) Conventional flow cytometry gating strategy. Cells were plotted forward versus side scatter using side scatter on a log scale, a large gate was drawn excluding debris. Cells were then plotted for either CD3 or CD14 versus forward scatter area to identify CD3^+^ T cell and CD14^+^ monocytes. CD3^+^ and CD14^+^ gated cells were finally plotted forward scatter (FS linear) versus side scatter (SSC log scale). Regions were drawn to identify SSC low and hi cells based on the negative controls. (**B**) Correlation of mean DF‐BDI (vertical axis) and mean SSC (horizontal axis) measurements of the CD14^+^ staining fraction of PBMC, Pearson correlation *P* ≤ 0.001, *r* = 0.978 and (**C**) correlation of mean DF‐BDI and mean SSC measurements of the CD3^+^ staining fraction of PBMC, Pearson correlation *P* ≤ 0.001, *r* = 0.879, *n* = 4 subjects. [Color figure can be viewed at wileyonlinelibrary.com]

**Figure 3:**
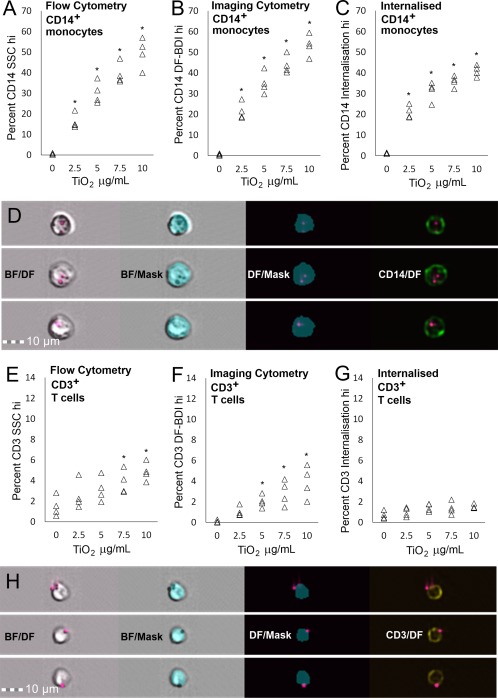
Conventional and imaging flow cytometry quantitation of monocyte and T lymphocyte association with TiO_2_. (**A**) Percentage of CD14^+^ monocytic cells gated as SSC intensity hi (**B**) DF‐BDI hi or (**C**) with internalized TiO_2_ with increasing dose in PBMC of *n* = 4 subjects. (**D**) Representative images of internalization hi gated CD14^+^ cells with the eroded brightfield internalization mask shown in brightfield, and in conjunction with CD14 and darkfield. (**E**) Percentage of CD3^+^ T cells gated as SSC intensity hi or (**F**) DF‐BDI hi and (**G**) with internalized TiO_2_ with increasing dose in PBMC of *n* = 4 subjects. (**H**) Example images of cell surface adherence on Internalization low DF‐BDI hi gated CD3^+^ T cells within PBMC and the eroded brightfield internalization mask shown in brightfield and in conjunction with CD3 and darkfield. *Significant difference from the control (*P* ≤ 0.05). [Color figure can be viewed at wileyonlinelibrary.com]

Both CD14^+^ gated monocytes and CD3^+^ gated T lymphocyte populations within PBMCs displayed dose‐dependent increases in mean darkfield bright detail intensity (DF‐BDI, as measured by imaging flow cytometry) and mean SSC, measured by conventional flow cytometry. An example of the gating strategy for conventional flow cytometry based on methods reported by Zucker et al. 18 is shown in Figure 2A. Correlation between mean DF‐BDI and SSC measurements indicated that both flow cytometric techniques similarly identified TiO_2_ cellular association with both monocytic and, surprisingly, a small but significant amount of the T‐lymphocyte population within PBMCs (Fig. 2B,C). Imaging flow analysis, which additionally allowed the quantitation of particle internalization to be determined, showed that the percentage of CD14^+^ cells with internalized TiO_2_, albeit lower, roughly mirrored TiO_2_ associated with CD14^+^ cells (Fig. 3A,C). The darkfield scattered light signals of TiO_2_ particles appeared as small punctate clusters within the CD14^+^ monocytes (Fig. 3D), as would be expected of particles grouped together inside endo‐lysosomal compartments within the cells, after active particle uptake by phagocytic cell types 29, 30, 31.

The same comparative analysis was performed on the CD3^+^ T‐lymphocyte populations within PBMC. Small but dose dependant increases in TiO_2_ association were also observed. However, CD3^+^ lymphocytes revealed a lack of TiO_2_ internalization and, instead, cell surface membrane adherence of particles, indicating that TiO_2_ internalization is limited to the phagocytosing CD14^+^ monocytic cells within PBMC (Fig. 3E–H).

**Figure 4:**
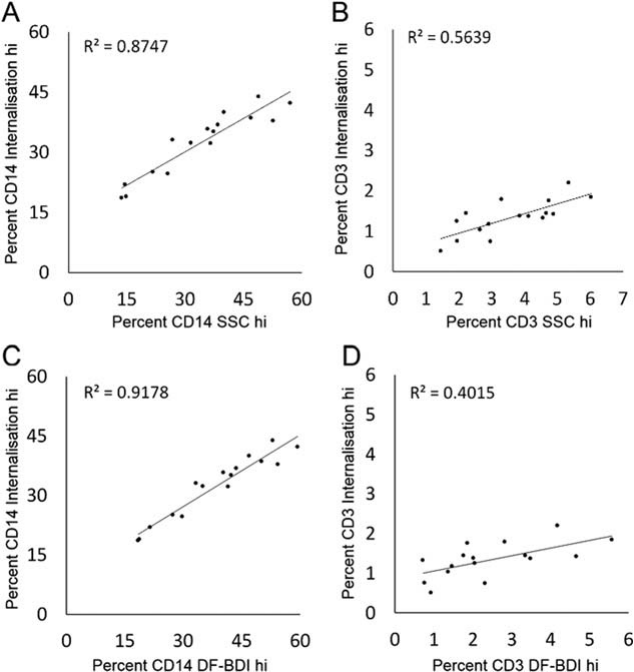
Correlation between internalization, darkfield‐bright detail intensity and side scatter for the identification of cellular TiO_2_. Correlation between (**A**) CD14^+^ cells gated as internalization hi and side scatter (SSC) hi; Pearson correlation *P* ≤ 0.001, *r* = 0.935, (**B**) CD3^+^ cells gated as internalization and SSC hi; Pearson correlation *r* = 0.750, (**C**) CD14^+^ cells gated as internalization and DF‐BDI hi; Pearson correlation *P* ≤ 0.001, *r* = 0.958, and (**D**) CD3^+^ cells gated as internalization and darkfield bright detail intensity (DF‐BDI) hi, Pearson correlation *r* = 0.633.

To interrogate the data further, we examined the degree of correlation between internalization and SSC or DF‐BDI measurements, which confirmed that internalization correlated to a high degree with both SSC and DF‐BDI measurements for CD14^+^ phagocytic cells, (Fig. 4A,C) but not CD3^+^ lymphocytes (Fig. 4B,D). This exemplified differences in particle interactions between phagocytic and non‐phagocytic immune cell types present in the same incubations, despite both cell types exhibiting some measure of TiO_2_ positivity. We conclude that, like conventional flow cytometry, flow imaging cytometry equally and accurately quantifies TiO_2_ cell association. However, comparison of imaging and conventional flow cytometry data additionally showed that adhesion of TiO_2_ to cell surface membranes, securely bound despite washing steps, impedes the accurate quantification of TiO_2_ internalization by conventional flow cytometry for some cell types present in blood.

### TiO_2_ Adherence and Internalization by Neutrophils and Monocytes in Whole Blood

We next applied the dual analysis approach to the development of the more complex matrix of whole blood for an assay facilitating the direct ex vivo identification of TiO_2_ particle uptake in whole blood. Building on methods developed by Baumann et al. we applied whole blood particle incubations to the down‐stream identification of TiO_2_ association by SSC and DF‐BDI. Nanoparticle uptake by monocyte and neutrophil populations has been reported upon exposure of whole blood to functionalized fluorescent polystyrene nanoparticles 32. We sought to determine whether TiO_2_ was similarly internalized by these two phagocytic immune cell types in whole blood. The study by Baumann et al. suggested that longer incubations better identify neutrophil uptake of particles 32. Therefore, fresh whole blood obtained from healthy volunteers was incubated with TiO_2_ for 24 h, in the presence of sodium heparin (to prevent coagulation). Monocytes and neutrophils were identified by staining for CD14 and CD16b, respectively, at the end of the assay, immediately after red blood cell lysis.

**Figure 5:**
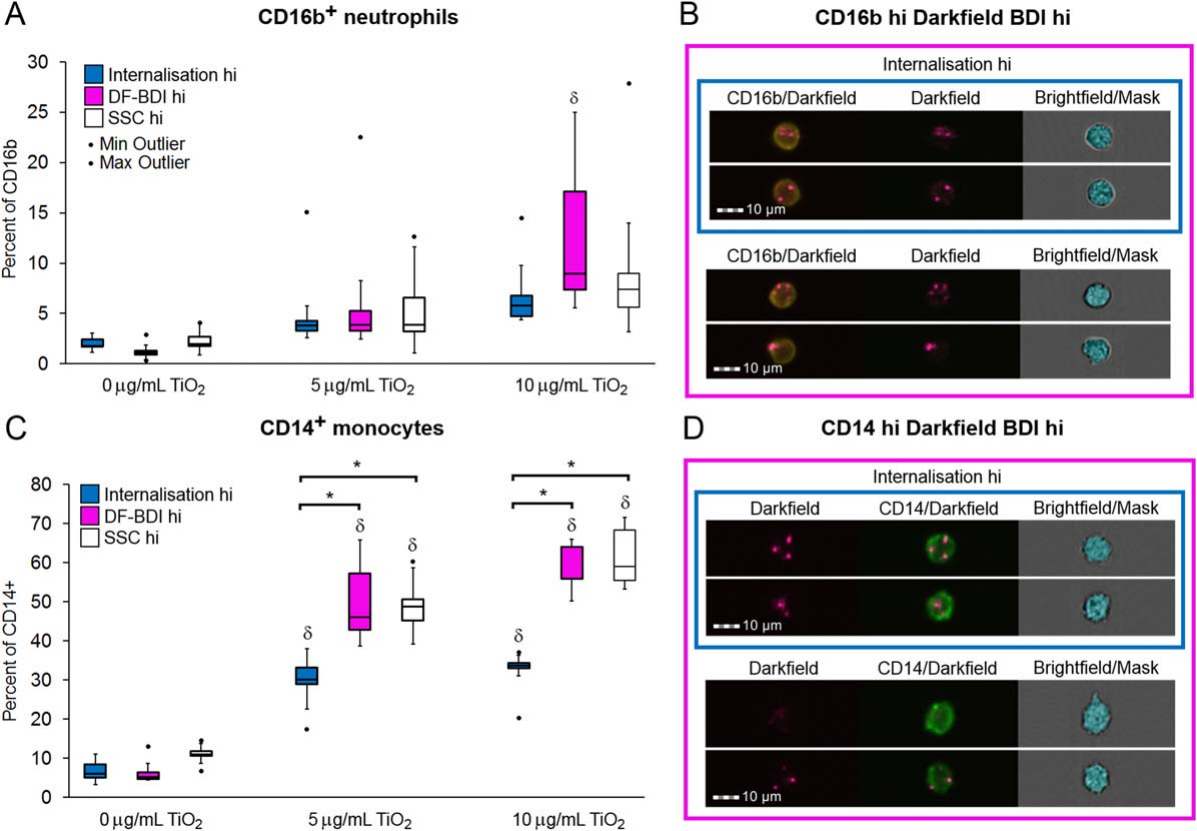
Neutrophil and monocyte association and internalization of TiO_2_ in whole blood by imaging and conventional flow cytometry. (**A**) Percentage of CD16b^+^ neutrophils gated as either internalization hi (blue), DF‐BDI hi (pink), or SSC hi (white) by flow imaging analysis (internalization/DF‐BDI) and flow cytometric analysis (SSC) after incubation with TiO_2_ in whole blood (*n* = 7 subjects). ^δ^Significant difference from the control. (**B**) Example imaging flow analysis images of CD16b^+^ (yellow) cells residing within the DF‐BDI hi and internalization gates, with the brightfield based internalization mask shown. (**C**) Percentage of CD14^+^ monocytes gated as either internalization hi (blue), DF‐BDI hi (pink) or SSC hi (white) by imaging flow analysis (internalization/DF‐BDI) and flow cytometric analysis (SSC) after incubation with TiO_2_ in whole blood (*n*   7 subjects). ^δ^Significant difference from the control and *significant differences between techniques as indicated (*P* ≤ 0.05). (**D**) Example analysis images of CD14^+^ (green) cells residing within the DF‐BDI hi and internalization gates, with the brightfield based internalization mask shown. Boxplots display Q1–Q3 with whiskers set at 1.5 × IQR (interquartile range) above the third quartile and 1.5 × IQR below the first quartile, minimum or maximum values that have fallen outside this range are shown as outliers (small black dots). [Color figure can be viewed at wileyonlinelibrary.com]

Identification of TiO_2_‐positive cells and TiO_2_ internalization were measured as described for the initial PBMC assays. Despite the highly granular nature of neutrophils (and so an increased capacity to scatter light), a significant increase in TiO_2_ association was still observed for CD16b^+^ neutrophils in incubations at the concentration of 10 μg/ml TiO_2_ as measured by DF‐BDI, and some of which was internalized TiO_2_ (Fig. 5A,B). High internalization and cell association of TiO_2_ by CD14^+^ monocytes were also observed (Fig. 5C,D). Multiple wash steps (×4) are required in preparation for the analysis of immune cells present in whole blood, so these data are likely to represent the minimum cell membrane adhesion of TiO_2_ by both monocyte and neutrophil populations upon exposure to TiO_2_ in a whole blood matrix.

**Figure 6:**
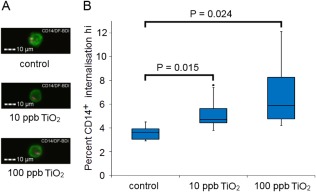
TiO_2_ internalization at physiological concentration by CD14^+^ monocytes in whole blood. (**A**) Example imaging flow analysis images of internalized TiO_2_ identified after incubation at low doses in one donor, CD14^+^ (green) and DF‐BDI (pink). (**B**) Percentage of CD14^+^ monocyte population with internalized DF‐BDI hi profile by imaging flow analysis after incubation with the low concentrations of 0–100 ppb (0–0.1 μg/ml) TiO_2_ in whole blood (*n* = 6 subjects), boxplots display Q1–Q3 with whiskers set at 1.5 × IQR (interquartile range) above the third quartile and 1.5 × IQR below the first quartile, minimum or maximum values that have fallen outside this range are shown as outliers (small black dots). Significant difference from the control is shown. [Color figure can be viewed at wileyonlinelibrary.com]

Finally, in order to confirm that the flow imaging cytometric assay possessed sufficient sensitivity to apply to the ex vivo analysis of blood after in vivo TiO_2_ exposures (such as ingestion of TiO_2_), fresh whole blood obtained from healthy volunteers was incubated as before, but with the reduced TiO_2_ concentration range of 0–100 ppb (0–0.1 μg/ml) that incorporated the low concentration levels of TiO_2_ particles reported in human blood after oral exposure 7. As observed previously, 24 h incubation with TiO_2_ resulted in increased mean DF‐BDI and internalized TiO_2_ was observed in association with CD14^+^ monocytic cells, shown in Figure 6. The data imply that the superior phagocytic function of monocytes, combined with the high refractive index of pigment grade TiO_2_, allows the identification of phagocytosed TiO_2_ present within resident blood monocytes after exposure at the low levels of 10 ppb (0.01 μg/ml) reported to reach the blood stream upon oral TiO_2_ exposure. In these experiments a minimum of 30,000 events were collected in order to keep acquisition times reasonable but this could be increased, which would in turn further increase the sensitivity of the assay. These experiments were performed in whole blood derived from healthy individuals with no anticipated immune stimulus present. There might be different rates of adherence or internalization of TiO_2_ in the presence of inflammatory signals in a disease setting.

## Discussion

We report an imaging flow technique, which allows the extent, cell specificity, and localization of TiO_2_ particle association to be measured at the single cell level in mixed populations of cells from whole blood. This technique, which combines conventional fluorescent staining for phenotypic markers, darkfield reflectance, brightfield, and the use of custom cell masking, exceeds the capacity of standard flow cytometry due to its ability to identify the cellular localization of TiO_2_ particles and has much greater speed and cell segmentation capability than microscopy. As such, it is ideal for quantitative analysis of single cells in complex mixed cell populations in terms of their interactions with TiO_2_. Interestingly, the imaging flow technique detected low levels of “reflectance‐positive” cells in all of the donor's baseline samples (i.e., no added TiO_2_). This is likely to be genuine background TiO_2_ as implied by prior ICP‐MS analyses of baseline human blood samples 7. Food, toothpaste, nutraceuticals, and pharmaceutical products account for the average UK adult ingesting around 3 mg TiO_2_ per day 6, some of which reaches the blood stream 7, 8. Hence, due to widespread daily human exposure to TiO_2_ it is likely that baseline blood samples will always have a degree of ‘natural background’ signal, so it is difficult to ascertain with certainty where the limit of detection exists for imaging flow cytometry. Moreover, advances in the field of darkfield microscopy in terms of resolution 21 are yet to be fully integrated with flow cytometric imaging techniques, so very small particles of TiO_2_ are likely to be undetectable currently. Nonetheless, flow imaging based assays, similar to conventional flow cytometric assays, are able to incorporate additional read outs such as staining for markers of immune activation and function thereby allowing direct associations to be made between TiO_2_ interactions with single cells (adhesion or internalization) and the functional consequences of such interactions on the *same* cell. Like others, we found imaging cytometry a powerful technique for the analysis of particle localization, this was dependant on tailoring custom masks in order to obtain an accurate analysis 9, 10, 11, 12, 13, 14, 15. There is always potential for some particles analyzed to be attached externally to the front or back of the cells, the occurrence of false positive internalization events is random and at the same frequency within all data files as it is dependent only on the orientation of the cells as they are imaged. Notwithstanding, imaging cytometry measurements of internalization are based on the objective automated analysis of images of hundreds or thousands of cells, with conclusions based on statistically significant differences between replicates of large populations, making them advantageous and significantly robust despite the potential for occurrence of random false events.

The identification of TiO_2_ using darkfield measurements was applied to whole blood assays, in order to obtain a closer representation of particulate interactions in vivo and to allow future ex vivo study of TiO_2_ blood cell interactions after in vivo exposure studies. This approach revealed neutrophil internalization of TiO_2_. Neutrophil interactions with nanoparticulate TiO_2_ have been shown to result in neutrophil activation in a proinflammatory fashion both in vitro 33 and in vivo 34. The precise TiO_2_–cellular interactions that result in this activation, and whether such effects extend to pigment grade TiO_2_ as well, should now be investigated 35, 36, 37. Studies examining TiO_2_ particle interactions with skin cells have reported adherence of TiO_2_ particles to cell membranes 38. Similarly, dual flow cytometry and imaging analyses also revealed considerable amounts of TiO_2_ membrane adherence by phagocytic and nonphagocytic immune blood cell types, which may act as a contributing factor to TiO_2_ uptake for the former, and/or the modulation of immune responses for both cell types.

Overall, imaging flow quantification of TiO_2_ particle–cell interactions in whole blood suggests that neutrophil–particle interactions, alongside the consequence of adherence of particles to cell surface membranes of nonphagocytic cell types, should also be considered in addition to the anticipated monocyte/macrophage uptake of TiO_2_ particles.

## Competing Interests

The authors declare they have no competing interests.

## Supporting information

Supporting File 1

Supporting File 2

Supporting File 3

Supporting File 4

Supporting MIFlowCyt
