# Supplementary material for: Imaging flow cytometry assays for quantifying pigment grade titanium dioxide particle internalization and interactions with immune cells in whole blood
Source: Cytometry A. 2017 Sep 20;91(10):1009–20. doi: 10.1002/cyto.a.23245 (PMC5698724; doi:10.1002/cyto.a.23245)
Supplement: Supplementary file 1 — Supporting File 1 [file CYTO-91-1009-s001.docx]

Imaging Flow Cytometry Assays for Quantifying Pigment Grade Titanium Dioxide Particle Internalisation and Interactions with Immune Cells in Whole Blood.

Rachel E. Hewitt, Bradley Vis, Laetitia C. Pele, Nuno Faria and Jonathan J. Powell.

**Additional File 1. TiO_2_ particle size characterisation.**


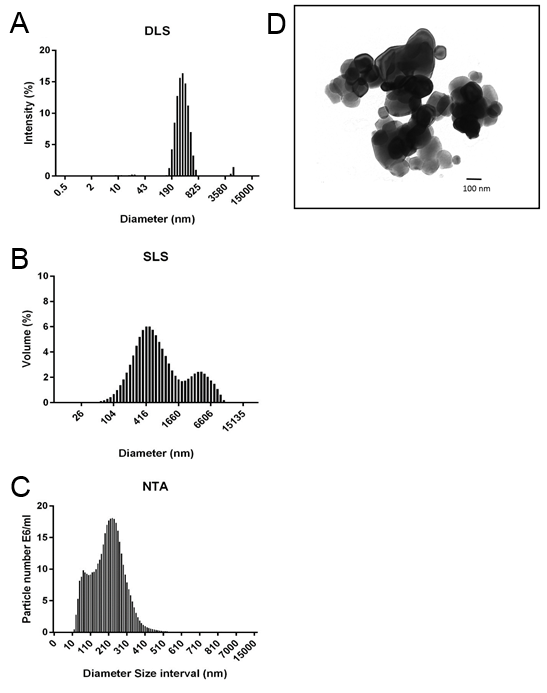


**Additional Figure 1.** **Food grade titanium dioxide particle characterisation.** Following dilution to 5µg/ml in supplemented TCM, TiO_2_ particles were analysed for particle size and size distribution immediately after preparation by **A**-dynamic light scattering (DLS), **B**-static light scattering (SLS) and **C**-nanoparticle tracking analysis (NTA). Data are represented as percentage volume (Volume %), percentage intensity (Intensity %) and particle number 10^6^ particles per millilitre (particle number E6/mL) as per manufacturer’s instructions. **D**- Particle size and size distribution of TiO_2_ powder analysed by transmission electron microscopy (TEM).
