# Supplementary material for: Imaging flow cytometry assays for quantifying pigment grade titanium dioxide particle internalization and interactions with immune cells in whole blood
Source: Cytometry A. 2017 Sep 20;91(10):1009–20. doi: 10.1002/cyto.a.23245 (PMC5698724; doi:10.1002/cyto.a.23245)
Supplement: Supplementary file 2 — Supporting File 2 [file CYTO-91-1009-s002.docx]

Imaging Flow Cytometry Assays for Quantifying Pigment Grade Titanium Dioxide Particle Internalisation and Interactions with Immune Cells in Whole Blood.

Rachel E. Hewitt, Bradley Vis, Laetitia C. Pele, Nuno Faria and Jonathan J. Powell.

**Additional File 2. Cell viability and TiO_2_ toxicity of neutrophils and monocytes in fresh whole blood assays.**

Whole blood assays were carried out as described in the main text of the paper. In brief, at the end of the 24 h incubation period red blood cells were removed by incubation with BD Pharm Lyse solution. After the 2nd wash, a final wash step was performed using tissue culture grade dPBS, the cell pellet re-suspended in a small volume of dPBS for viability staining on ice in the dark for 15 min (1μL per test, live/dead Violet, Invitrogen L34955). Cells were then washed with cold dPBS 1% BSA and stained for 20 min on ice in the dark in a small volume of cold PBS/BSA (facs wash buffer) and the appropriate amount of antibody staining mix (anti-human CD14 and CD16b). After a final wash with cold dPBS/BSA cell were re-suspended the in a small volume of cold 2% PFA. Cells were acquired immediately on either a Cyan-ADP flow cytometer using Summit software for acquisition and analysis (Beckman Coulter) using SSC on a log scale with increased threshold to prevent acquisition of residual dying red blood cells, acquiring a minimum of 400,000 events per sample and the Imagestream X, acquiring a minimum of 50,000 events.

Additional figure 2 shows the viability of CD14^+^ monocytes and CD16b^+^ neutrophils after 24 h incubation with or without titanium dioxide particles gated in the same way as the analyses described in the manuscript. We found that there was no significant death of cells during the assay and that CD14^+^ monocytes and CD16b^+^ viability was very good in fresh whole blood assays up to 24 h, as has been reported at the low doses used in these experiments (26-27). The minimal manipulation of the whole blood and the cells during the assay period was also very likely to contribute to the low levels of death within the cell populations examined.


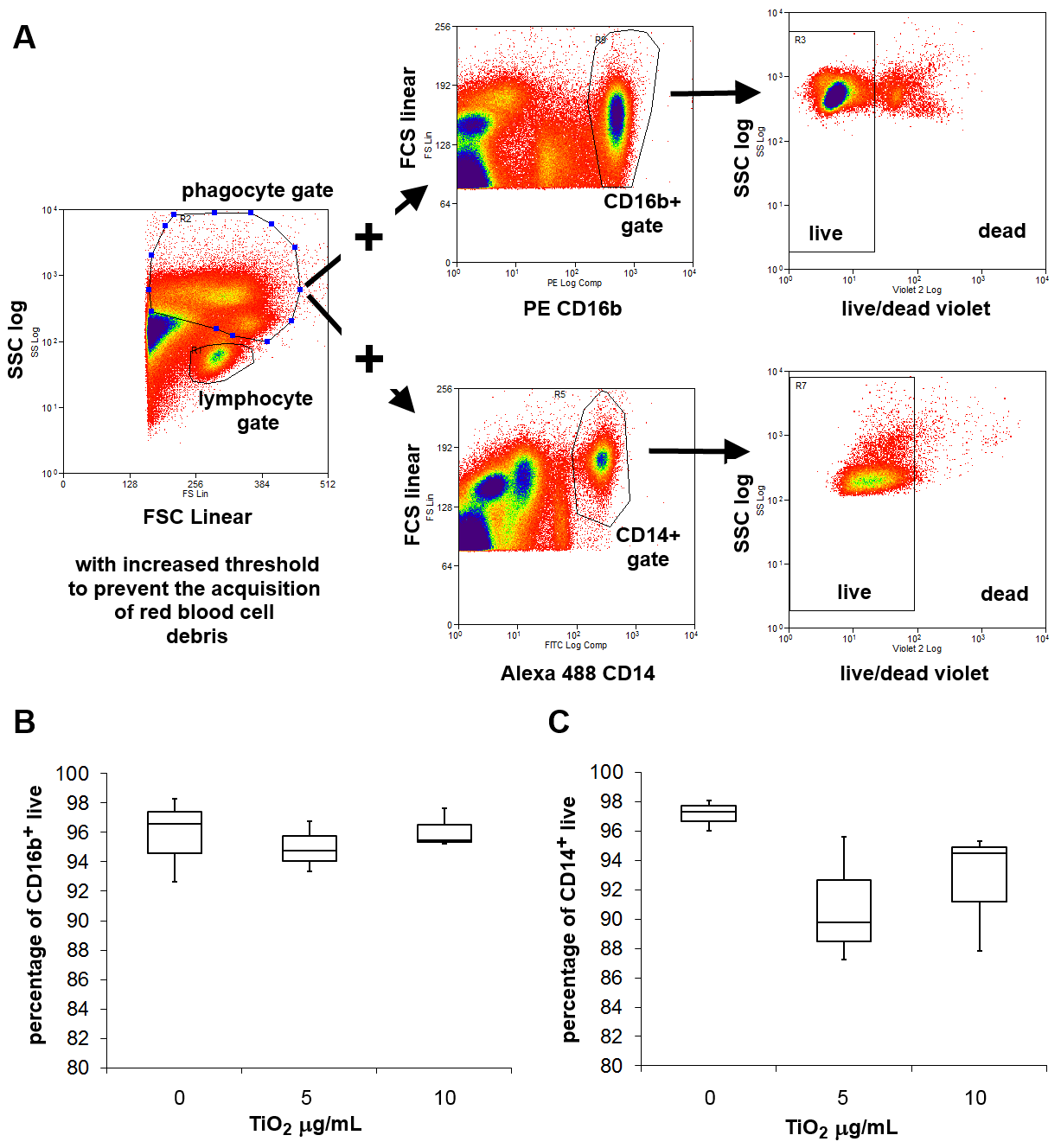


**Additional figure 2. Viability of CD14^+^ monocytes and CD16b^+^ neutrophils after 24 h incubation with or without titanium dioxide particles. A.** Gating strategy for flow cytometric analysis of CD16b^+^ neutrophils and CD14^+^ monocytes. Representative dot-plots of whole blood after red blood cell lysis and flow cytometric staining panel. Cells were first plotted forward versus side scatter using side scatter on a log scale, phagocyte and lymphocyte gates were drawn excluding red blood cell debris. Cells were then plotted for either CD16b or CD14 versus forward scatter area to identify CD16^+^ neutrophils and CD14^+^ monocytes. Viability of the CD16b^+^ and CD14^+^ gated cells were then assessed using the dead cell stain live/dead violet which positively identifies dead or dying cells by increases in fluorescence due to increased staining of cells with compromised cell membrane integrity. **B**. Boxplots displaying viable CD16b^+^ gated neutrophils after 24 h whole blood incubations (n = 3), and **C**. viable CD14^+^ gated monocytes after 24 h whole blood incubations (n = 3). Boxplots display Q1-Q3 with whiskers set at 1.5 x IQR (interquartile range) above the third quartile and 1.5 x IQR below the first quartile, i.e. the minimum and maximum values within this range.
