# Supplementary material for: Imaging flow cytometry assays for quantifying pigment grade titanium dioxide particle internalization and interactions with immune cells in whole blood
Source: Cytometry A. 2017 Sep 20;91(10):1009–20. doi: 10.1002/cyto.a.23245 (PMC5698724; doi:10.1002/cyto.a.23245)

Imaging Flow Cytometry Assays for Quantifying Pigment Grade Titanium Dioxide Particle Internalisation and Interactions with Immune Cells in Whole Blood.

Rachel E. Hewitt, Bradley Vis, Laetitia C. Pele, Nuno Faria and Jonathan J. Powell.

**Additional File 3. TiO_2_ localisation and association with monocyte and lymphocyte populations within PBMC.**

For imaging flow analysis, area (size of the masked cells in square microns) versus aspect ratio (the ratio of the minor axis divided by the major axis) of the brightfield cell images were used to create an initial dot plot to identify PBMC populations of interest as well as doublet and debris exclusion. A representative plot and image examples are shown in additional figure 3A. From the single cell gate (A) further gates are drawn, starting with cells in best focus (using gradient RMS, which measures the sharpness quality of an image) shown in additional figure 3B, followed by gating on fluorescence positives using fluorescence intensity histograms for CD14^+^ (ch2, additional figure 3C) and CD3^+^ (ch5, additional figure 3D) populations within PBMC. CD14^+^ or CD3^+^ (single, focused) gated cells were then plotted as scatter plots using bright detail intensity (BDI) measurements for brightfield (vertical axis) and darkfield (horizontal axis). The bright detail intensity (BDI) feature computes the intensity of localised bright spots within the masked cell area of the image. In this analysis BDI R3 was used, which computes the intensity of bright spots that are 3 pixels in radius or less. BDI scatter plots of the gated CD14^+^ or CD3^+^ cells allowed a region to then be drawn selecting TiO_2_ positive cells, identified by increased BDI darkfield measurements. These are shown in additional figure 3E for CD14^+^ and additional figure 3G for CD3^+^ gated cells as darkfield (DF) positive, representative image examples of the cells residing within the DF positive and DF negative gates are also shown.

The measurement of internalised TiO_2_ darkfield signals relies on combining the application of two imaging cytometry features. Firstly, imaging cytometers use a microscope objective to collect transmitted light, scattered light and emitted fluorescence (28). At the standard 40X objective used in these assays this provides a standard cross sectional image of each cell with a 4 μm depth of focus (for the PBMC titration experiments an extended depth of field was applied giving 16 μm focal range which allowed crisper fluorescent imaging throughout the depth of the cell image, but this is not necessary for a cross sectional image). This cross sectional representation of each cell allows the definition of the cell surface membrane at the outer perimeter of the cell image, as can be seen in additional figure 3 where the location of cell surface associated CD14 (monocytes) and CD3 (T lymphocytes) can be seen. This cross sectional image can then be utilised to create an internalisation mask, which provides information on a specific area of interest. For the measurement of internalisation the area of interest is the area of the cell excluding the outer cell membrane and so an internalisation mask was created eroded by 4 pixels from the outer edge of the cell brightfield image. Each pixel has a size of 0.5 μm so creating an area of interest up to the equivalent to 2 μm in from the cell surface membrane. This excludes darkfield signals associated with TiO_2_ at the cell surface, internalisation masks once applied are unique to every cell, examples can be seen on the image examples shown in additional figure 3F. Measurement of the percentage of CD14^+^ (or CD3^+^) population with internalised TiO_2_ particles identified by increased darkfield BDI measurements are defined using the internalisation feature (defined as the ratio of intensity inside the cell to the intensity of the entire cell) and assigned an internalisation score, with higher scores indicating internalisation. Histogram plots of internalisation scores for Ch6 (Darkfield/SSC) were used to create internalisation hi and low gates within the CD14^+^ and CD3^+^ gated populations shown in additional figure 3F and 3H).

**Additional Figure 3. TiO_2_ localisation and association with monocyte and lymphocyte populations within PBMC.** **A**. Area (size of the masked cells in square microns) versus aspect ratio (the ratio of the minor axis divided by the major axis) of the brightfield cell images are used to create an initial dot plot to identify cell populations of interest exclude doublets and debris. Single cells in best focus (using gradient RMS) were then gated as shown in **B**, followed by gating on fluorescence positives through fluorescence intensity histograms for CD14^+^ cells in Ch 02 **C**, and CD3+ cells in Ch 05 **D**. Single, focused gated cells were then plotted as scatter plots using bright detail intensity (BDI) measurements for brightfield (vertical axis) and darkfield (horizontal axis). The bright detail intensity (BDI) feature computes the intensity of localised bright spots within the masked cell area of the image, BDI R3 used in these analyses computes the intensity of bright spots 3 pixels in radius or less. BDI scatter plots of the gated CD14^+^ or CD3^+^ cells allowed a region to then be drawn selecting TiO_2_ positive cells, identified by increased BDI darkfield measurements shown in **E** for CD14^+^ gated cells, and **G** for CD3+ gated cells (DF positive). Representative image examples of the cells residing within gates are shown. Measurement of the percentage of CD14^+^ and CD3^+^ populations with internalised TiO_2_ particles identified by increased darkfield BDI measurements were defined using the internalisation feature and assigned an internalisation score. Histogram plots of internalisation scores for Ch6 (Darkfield/SSC) were used to create internalisation hi and low gates within the CD14^+^ and CD3^+^ gated populations shown in **F** for CD14^+^ cells and **H** for CD3^+^ cells.


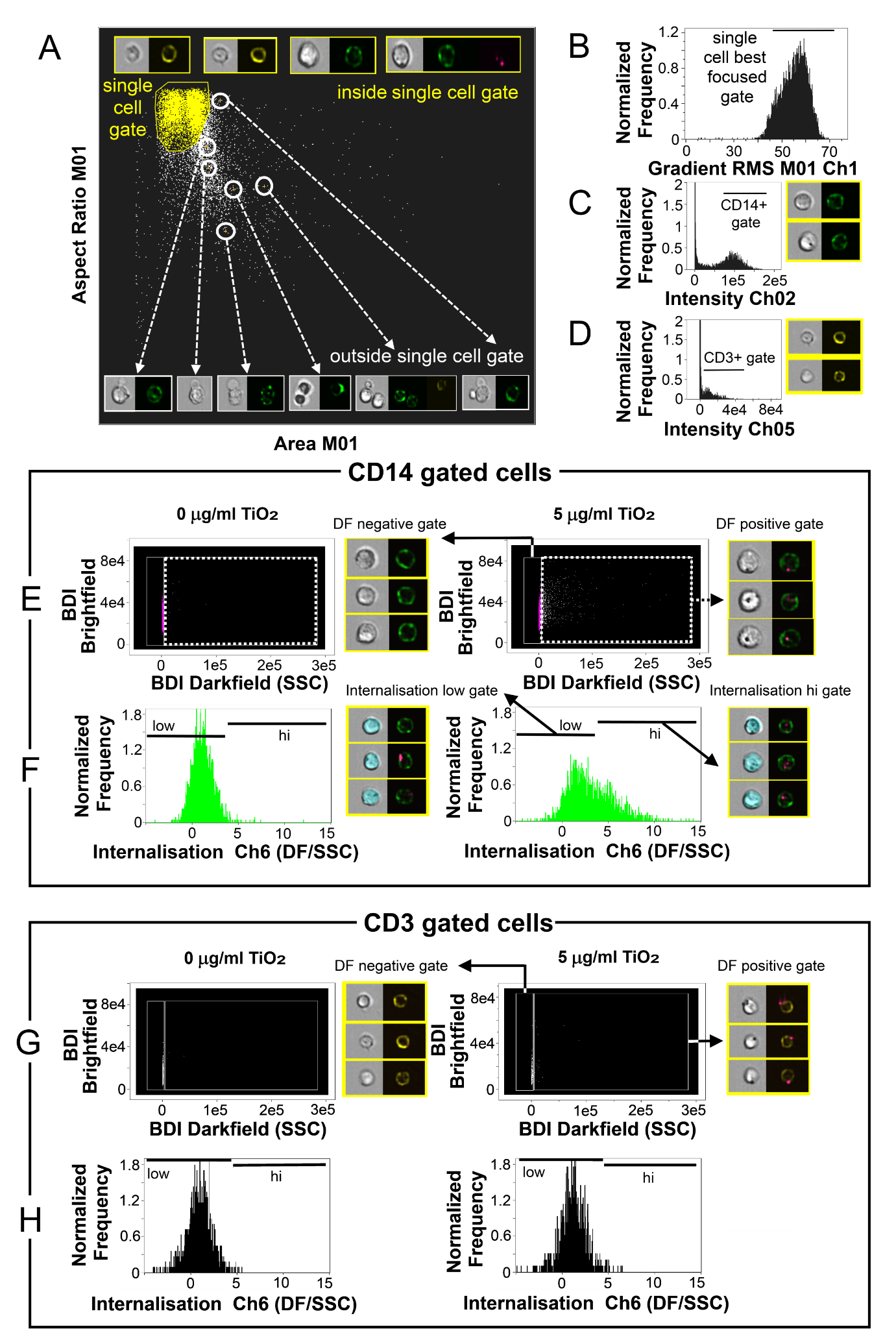

Supplement: Supplementary file 3 — Supporting File 3 [file CYTO-91-1009-s003.docx]
