# Supplementary material for: Imaging flow cytometry assays for quantifying pigment grade titanium dioxide particle internalization and interactions with immune cells in whole blood
Source: Cytometry A. 2017 Sep 20;91(10):1009–20. doi: 10.1002/cyto.a.23245 (PMC5698724; doi:10.1002/cyto.a.23245)
Supplement: Supplementary file 4 — Supporting File 4 [file CYTO-91-1009-s004.docx]

Imaging Flow Cytometry Assays for Quantifying Pigment Grade Titanium Dioxide Particle Internalisation and Interactions with Immune Cells in Whole Blood.

Rachel E. Hewitt, Bradley Vis, Laetitia C. Pele, Nuno Faria and Jonathan J. Powell.

**Additional File 4. Cell diameter and correlation with Darkfield signals in the presence of TiO_2_.**

PBMC incubations with increasing amounts of TiO_2_ were carried out as described in the main text of the paper. Cell staining and flow cytometric imaging were carried out as detailed in the methods section. For measurement of cell diameters, the same single cell, best focused, followed by CD14 and CD3 gates used in the main analysis were used as detailed in the main text and additional file 3. For the measurement of cell diameters, a tight object mask based on the brightfield cell images was created and utilised to measure cell diameters using the IDEAS diameter feature. This feature provides the diameter of the circle that has the same area as the object (in this case all object masked cells). It was possible to utilise this feature effectively as the cells examined are all circular in shape and a tight (cell hugging mask) was used. The cell diameters of all CD14^+^ and CD3^+^ gated cells were first examined after exposure to increasing concentrations of TiO_2_. The analysis was then further extended to specifically examine only the cell diameters of CD14^+^ cells which displayed hi bright detail intensity of darkfield fluorescence (CD14^+^ BDI DF hi).

Additional figure 4 shows the mean cell diameters of CD14^+^ (additional fig 4D) and CD3^+^ (additional fig 4 E) cells measured after exposure to increasing concentrations of TiO_2_. CD3^+^ gated cells remained at a constant diameter of 9 – 9.5 microns irrespective of incubations with increasing concentrations of TiO_2_. Similarly, CD14^+^ gated cells remained at a constant diameter of ~12 microns irrespective of incubation with increasing concentrations of TiO_2_, as anticipated no cell swelling appeared to occur in response to exposure TiO_2_ at these sub-toxic concentrations (26-27). We then further established that the increases in bright detail intensity of darkfield fluorescence (CD14^+^ BDI DF^+^) associated with exposure to increasing concentrations of TiO_2_ particle and CD14^+^ monocytic internalisation of TiO_2_ (figures 3 and 4) were not an artefact of cell swelling and increased scattered light. Mean diameters of CD14^+^ gated cells residing within the darkfield positive gate (see figure 1 of the main text for an example) were examined for correlation with mean darkfield bright detail fluorescence intensities (of the same cells). No correlation was observed between cell diameter and mean bright detail intensity in the darkfield channel (BDI DF) for CD14^+^ cells residing within the darkfield positive gate (CD14^+^ BDI DF^+^). Furthermore, the diameter of these CD14^+^ BDI DF^+^ cells fell well within the normal diameter range observed for CD14^+^ cells irrespective of the presence of TiO_2_. The observation that no changes in cell diameter were observed in response to TiO_2_ exposure (for either CD14^+^ or CD3^+^ cells), coupled with no correlation between darkfield BDI and cell diameter measurements, led us to conclude that cell swelling or shrinking associated with toxic responses and cell death were not responsible for the increases in darkfield BDI and side scatter measurements upon TiO_2_ exposure.


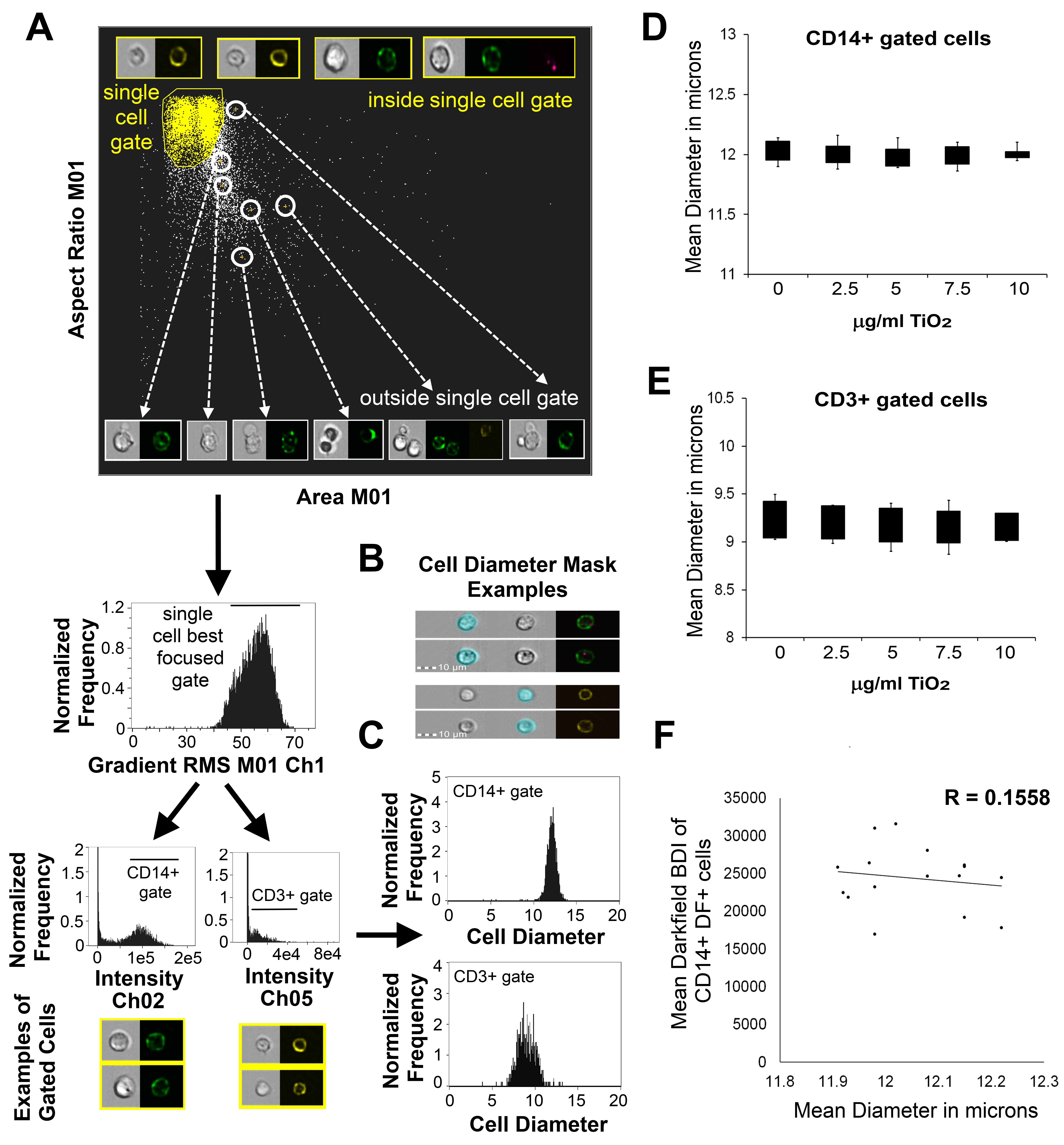


**Additional figure 4. Cell diameter and correlation with Darkfield signals in the presence of increasing concentrations of TiO_2_. A.** Gating strategy for flow imaging analysis of CD14^+^ monocytes and CD3^+^ lymphocytes. For data analysis, cells were first plotted area versus aspect ratio of the brightfield images and a single cell gate drawn, followed by a best focused gate (based on brightfield, gradient root mean squared feature values), followed by either CD14^+^ or CD3^+^ gates based on intensity of the respective fluorophore channels. A tight object mask based on the brightfield cell images was then created and applied to the measurement of cell diameters using the diameter feature, examples of the tight object mask applied are shown in **B**. Histograms of the cell diameters of the CD14 and CD3 gated cell populations were then drawn, examples are shown in **C**. Boxplots displaying cell diameters after exposure to increasing concentrations of TiO_2_ (data from n = 4 donors for each concentration) for CD14 gated **D.** and CD3 gated **E.** populations within PBMC. Boxplots display Q1-Q3 with whiskers set at 1.5 x IQR (interquartile range) above the third quartile and 1.5 x IQR below the first quartile, i.e. the minimum and maximum values within this range. **F**. Correlation between the mean darkfield bright detail intensity (BDI Darkfield) values for CD14^+^ gated cells residing within the darkfield positive gate (DF positive – see figure 2 for an example of this gate) with cell diameter measurements for the same (CD14^+^ BDI DF positive) cell population.
