# Supplementary material for: Imaging flow cytometry assays for quantifying pigment grade titanium dioxide particle internalization and interactions with immune cells in whole blood
Source: Cytometry A. 2017 Sep 20;91(10):1009–20. doi: 10.1002/cyto.a.23245 (PMC5698724; doi:10.1002/cyto.a.23245)
Supplement: Supplementary file 5 — Supporting MIFlowCyt [file CYTO-91-1009-s005.doc]

**Cytometry Part A**

**Author Checklist: MIFlowCyt-Compliant Items**

| **Requirement** | **Please Include Requested Information** |
| --- | --- |
| 1.1. Purpose | The development of an assay for the identification and quantification of TiO_2_ particle internalizing immune cells in whole blood, suitable for *ex vivo* assays after *in vivo* exposures and short term *in vitro* whole blood assays. |
| 1.2. Keywords | Flow cytometry, flow imaging, side scatter, darkfield, titanium dioxide, nanoparticles, sub-micron particles, internalisation, whole blood, peripheral blood mononuclear cells, neutrophils, monocytes, membrane adherence. |
| 1.3. Experiment variables | Human Peripheral Blood Mononuclear Cells (PBMC) and fresh whole heparinised blood samples were treated with TiO_2_ particles and increases in side scatter or darkfield bright detail intensity were measured in specific cell populations identified by phenotypic markers (CD3 for T lymphocytes, CD14 for monocytes and CD16b for neutrophils). |
| 1.4. Organization name and address | Biomineral Research Group  Dept. of Veterinary Medicine  University of Cambridge  Madingley Road  Cambridge CB3 OES  UK |
| 1.5. Primary contact name and email address | Dr Rachel Hewitt [reh63@cam.ac.uk](mailto:reh63@cam.ac.uk)  Rachel.Hewitt@mrc-ewl.cam.ac.uk |
| 1.6. Date or time period of experiment | 2015 - 2016 |
| 1.7. Conclusions | Association and cellular localisation of TiO_2_ particles can be accurately identified and quantified by darkfield reflectance flow imaging analysis in whole blood assays, enabling the measurement of TiO_2_ particle-immune cell interactions in blood and their consequence directly *ex vivo*. |
| 1.8. Quality control measures | The Imagestream X was fully calibrated and passed all tests directly prior to the acquisition of samples using the machines calibration and test scripts. The CyAn ADP flow cytometer alignment was calibrated with beads (Spherotech) prior to acquisition. |
| 2.1.1.1. (2.1.2.1., 2.1.3.1.) Sample description | Food Pigment Grade Titanium dioxide TiO2(s) (Anatase, Special AHR;Tioxide UK Ltd, Cleveland, UK. |
| 2.1.1.2. Biological sample source description | Human Peripheral Blood Mononuclear Cells (PBMC) obtained from Leucocyte Cones (purchased from the National Blood Service) and fresh whole heparinized blood samples obtained from consenting healthy volunteers. See text for further details. |
| 2.1.1.3. Biological sample source organism description | Human |
| 2.1.2.2. Environmental sample location | Food grade Titanium dioxide TiO_2_ Kowet anatase TiO_2_ (product No 03970) was obtained in powder form from Sensient Colors Inc. (St. Louis, MO, USA). |
| 2.3. Sample treatment description | PBMC in tissue culture media or fresh whole blood incubated with TiO_2_ particles for 3 or 24 hours. |
| 2.4. Fluorescence reagent(s) description | TiO_2_ particles are highly reflective and scatter light detectable by the SSC and darkfield detectors on flow cytometers and flow imagers respectively. See text for more details. T lymphocytes, monocytes and neutrophils within PBMC or whole blood were identified by staining for the phenotypic markers CD3, CD14 and CD16b (respectively) with the following conjugated antibodies; PE-Cy5 Mouse anti-human CD3, Clone UCHT1 (BD Biosciences 555334), Alexa fluor 488 Mouse anti human CD14, Clone M5E2 (BD Biosciences 557700) and PE Mouse anti human CD16b, Clone CLB-gran11.5 (BD Biosciences 550868). |
| 3.1. Instrument manufacturer | Beckman Coulter, Amnis Merck Millipore |
| 3.2. Instrument model | CyAn ADP flow cytometer, ImagestreamX Mark I |
| 3.3. Instrument configuration and settings | CYAn ADP: 3 lasers (405, 488, 642nm) in standard configuration and standard filter sets. Imagestream X in standard configuration equipped with 405 nm and 488 nm lasers for excitation and a 785 nm laser for a scatter signal with standard filter sets, multi magnification (20X/40X/60X) and extended depth of field. No alterations or changes had been made from the standard configuration for either machine. |
| 4.1. List-mode data files | All files are available upon request from the corresponding author. |
| 4.2. Compensation description | For Imagestream X flow imaging experiments raw data files were saved without compensation (rif files). A compensation matrix was generated from single stained cell samples (1 for each fluorophore used) data and applied using IDEAS V6.0 software (Amnis Merck Millipore) to create compensated data files (cif files) and data analysis files (daf files).  For flow cytometry experiments data files were saved without applying compensation. Spectral overlap was assessed and data analyzed with the use of unstained and single stained compensation cell samples (1 for each fluorophore used). Compensation was applied according to single stains during post-acquisition analysis to minimize spectral overlap using Summit V4.3 software (Beckman Coulter, UK). |
| 4.3. Data transformation details | Data were analyzed using IDEAS V6.0 software (Amnis Merck Millipore) and Summit V4.3 software (Beckman Coulter, UK) to create dot plot, density plots and histograms. |
| 4.4.1. Gate description | Gating strategy for Imagestream analysis of TiO2 association and internalisation.  Representative plots of PBMC from 1 donor. For imaging flow analysis, Area (defined as the size of the masked cells in square microns) versus Aspect ratio (the ratio of the minor axis divided the major axis) of the brightfield cell images are used to create an initial dot plot to identify cell populations of interest as well as doublet and debris exclusion. Representative plot and selected image examples are shown. From the single cell gate sequential gates are drawn, starting with cells in best focus (using gradient RMS, which measures the sharpness quality of an image through the enumeration of pixel values) shown in B, followed by gating on fluorescence positives through fluorescence intensity histograms for CD14+ in Ch 02 C, and CD3+ in Ch 05 D. Single, focused gated cells were then plotted as scatter plots using bright detail intensity (BDI) measurements for brightfield (vertical axis) and darkfield (horizontal axis). The bright detail intensity (BDI) feature computes the intensity of localised bright spots within the masked cell area of the image, BDI R3 used in these analyses computes the intensity of bright spots 3 pixels in radius or less. BDI scatter plots of the gated CD14+ or CD3+ cells allowed a region to then be drawn selecting TiO2 positive cells, identified by increased BDI darkfield measurements shown in E for CD14+ and G for CD3+ gated cells as darkfield (DF) positive. Representative image examples of the cells residing within gates are shown. Measurement of the percentage of CD14+ (and CD3+) population with internalised TiO2 particles identified by increased darkfield BDI measurements were defined using the internalisation feature and assigned an internalisation score. Histogram plots of internalisation scores for Ch6 (Darkfield/SSC) were used to create internalisation hi and low gates within the CD14+ and CD3+ gated populations shown in F for CD14+ cells and H for CD3+ cells). 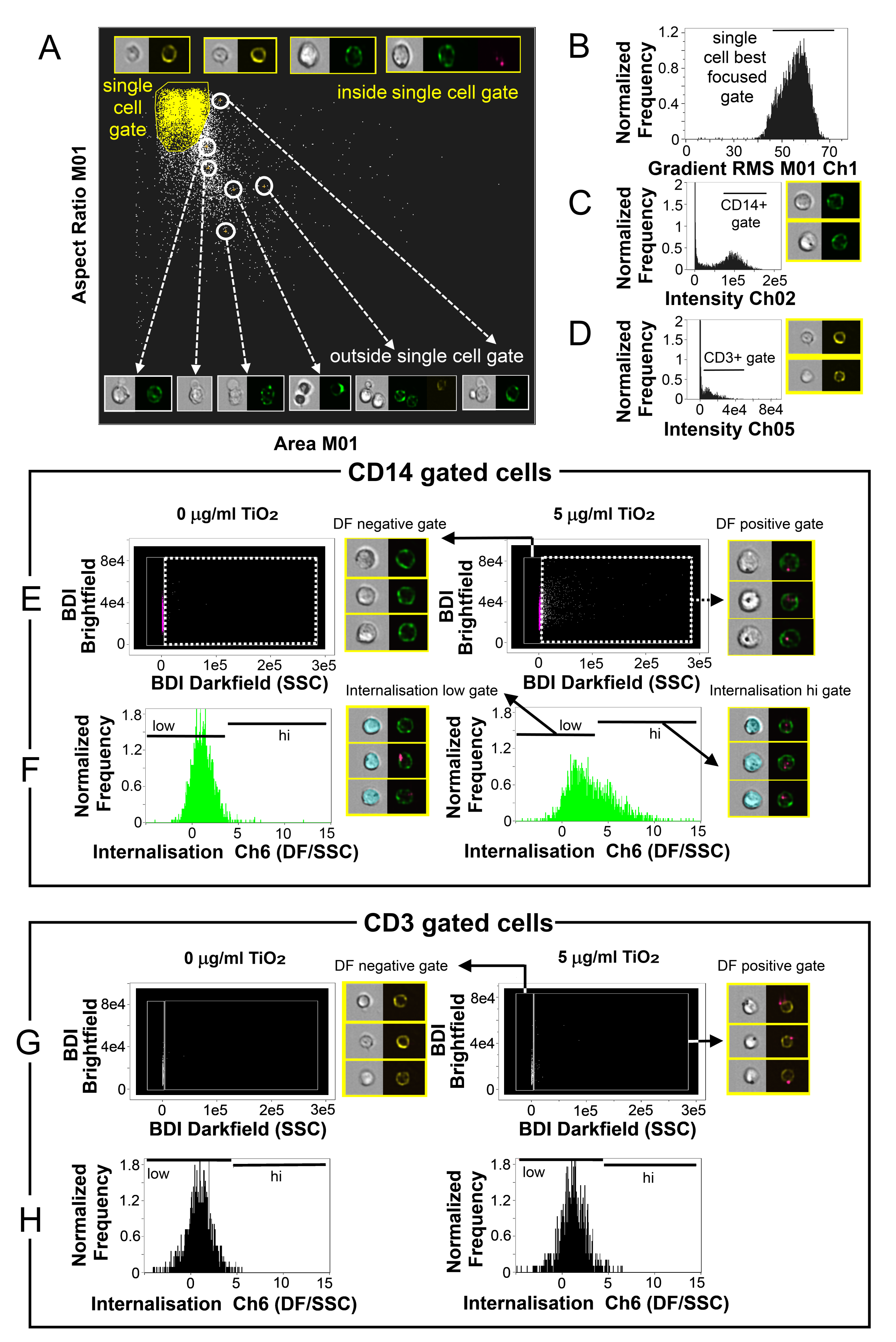  Gating strategy for flow cytometric analysis of TiO2 association. Representative dot-plots of PBMC from 1 donor. Conventional flow cytometry gating strategy. Cells were plotted forward versus side scatter using side scatter on a log scale, a large gate was drawn excluding debris. Cells were then plotted for either CD3 or CD14 versus forward scatter area to identify CD3+ T cell and CD14+ monocytes. CD3+ and CD14+ gated cells were finally plotted forward scatter (FS linear) versus side scatter (SSC log scale). Regions were drawn to identify SSC low and hi cells based on the negative controls.  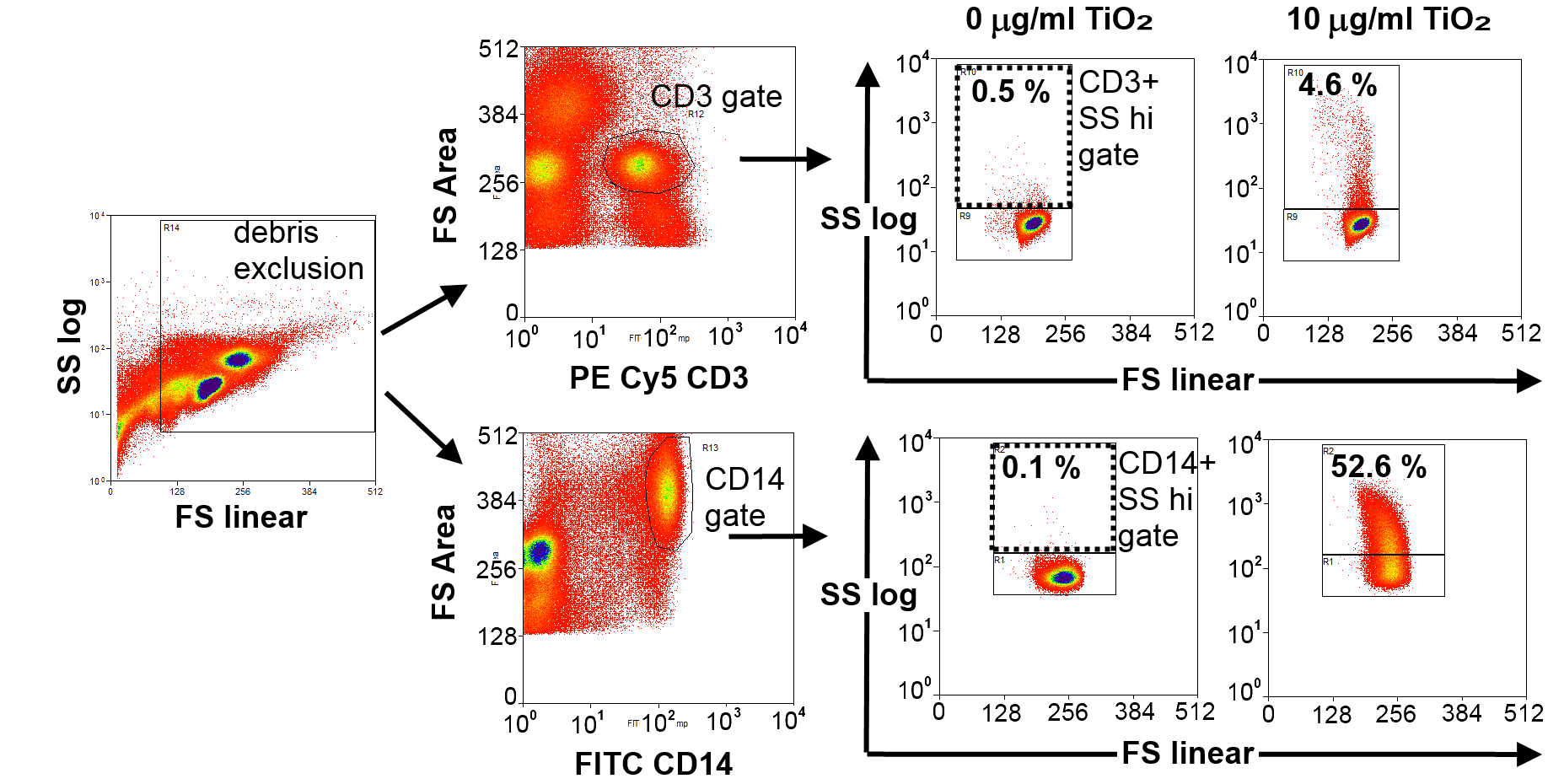 |
| 4.4.2. Gate statistics | For the measurement of Ti positive cells in PBMC or RBC lysed whole blood, mean flurorescence intensity measurements were used for the entire gated CD3^+^, CD14^+^ or CD16b^+^ population was used. For the percentage of Ti positivity within gated populations, the percentage of the entire gated population was used e.g. percent CD3^+^ cells residing within the Ti hi gate was used. |
| 4.4.3. Gate boundaries | Gate boundaries were based on the exclusion of cell debris, clusters of positively staining cell populations and changes in SSC or darkfield values compared to baseline (negative control) values. |

**Notes**

Feel free to use more space than allocated.

You can embed graphics/figures in this document, if needed.

Please make sure to save the document in Microsoft Word version 2003 or older, before uploading to ScholarOne Manuscripts. When uploading this checklist to ScholarOne Manuscripts, please choose the “Supplementary Material for Review” category.

Please note that if your paper is accepted, the checklist will be published as an Online Supporting Information.

For any questions, please contact the Cytometry Part A editorial office at [Cytometrya@wiley.com](mailto:Cytometrya@wiley.com).
